# 

**Published:** 1867-02

**Authors:** 


					﻿DENTAL MEETING.
Louisville, Ky., March 1st, 1867.
Dear' Sir: — The Third Semi-Annual Meeting of the
Central States Dental Association will be held in the
City of Memphis, Tenn., commencing on Tuesday the 9th
of April, 1867, at 10 o'clock, a. m.
Your attendance is earnestly requested.
W. H Shadoan,
Secretary.
Of Rental Register
OF THE WEST.
Issued Monthly, ,at $3 a Year in Advance.
DEVOTED TO THE INTERESTS OF THE DENTAL PROFESSION.
EDITED BY J. TAFT AND G. WATT.
The volume commences in January and closes in December.
The Register being issued monthly is always fresh, therefore indispensable to the practi-
tioner who desires to keep posted up with the new inventions and improvements which are
daily developed. Neither expense nor effort will be spared to give value and variety to its
contents. Articles will be illustrated with well executed _ engravings whenever they will add
to the interest or assist in explanation.
Its extensive circulation and frequency of issue makes it one of the BEST DENTAL
ADVERTISING MEDIUMS in the United States.
RATES OF ADVERTISING.
One page, 1 year, $50. TTalf page, 1 year, $30. Quarter page, or card, 1 year $20.
All advertising bills payable in advance, or at furthest after the issue of the first number
containing the advertisement. All transient advertisements to be paid for in advance.
BS5" CONTRIBUTIONS SOLICITED.
Address, J. TAFT, or “DENTAL REGISTER,” Cincinnati, Ohio.
Business Communications to
-T. TAFT, ^Publisher,
No. 238 Fourth St., between Plum and Control Avenue.
				

